# Supplementary material for: Hot spot prediction in protein-protein interactions by an ensemble system
Source: BMC Syst Biol. 2018 Dec 31;12(Suppl 9):132. doi: 10.1186/s12918-018-0665-8 (PMC6311905; doi:10.1186/s12918-018-0665-8)
Supplement: Supplementary file 2 — All relevant MATLAB code. A simple Matlab implement of our predictor. (ZIP 19,742 kb) [file 12918_2018_665_MOESM2_ESM.zip › Additional file 2/Read ASA/ASA website/dbMPIKT/4P23D.html]

 NetSurfP - Protein Surface Accessibility and Secondary Structure Predictions

  

|  |  |  |
| --- | --- | --- |
|  |  | NetSurfP - Protein Surface Accessibility and Secondary Structure Predictions  Technical University of Denmark |

  


---

```
# For publication of results, please cite:
# A generic method for assignment of reliability scores applied to solvent accessibility predictions.
# Bent Petersen, Thomas Nordahl Petersen, Pernille Andersen, Morten Nielsen and Claus Lundegaard
# BMC Structural Biology 2009, 9:51 doi:10.1186/1472-6807-9-51
#
# Column 1: Class assignment - B for buried or E for Exposed - Threshold: 25% exposure, but not based on RSA
# Column 2: Amino acid
# Column 3: Sequence name
# Column 4: Amino acid number
# Column 5: Relative Surface Accessibility - RSA
# Column 6: Absolute Surface Accessibility
# Column 7: Z-fit score for RSA prediction
# Column 8: Probability for Alpha-Helix
# Column 9: Probability for Beta-strand
# Column 10: Probability for Coil
E G  4P23D                  1    0.843  66.320  -0.849   0.003   0.003   0.994
E G  4P23D                  2    0.440  34.628  -2.041   0.018   0.088   0.893
E S  4P23D                  3    0.354  41.512  -0.300   0.074   0.484   0.442
B L  4P23D                  4    0.154  28.142  -1.287   0.169   0.612   0.219
E V  4P23D                  5    0.316  48.631   0.386   0.268   0.505   0.227
E P  4P23D                  6    0.442  62.734  -0.078   0.373   0.401   0.226
B R  4P23D                  7    0.256  58.647   0.430   0.352   0.332   0.316
B G  4P23D                  8    0.175  13.741  -0.146   0.352   0.332   0.316
B S  4P23D                  9    0.223  26.147  -1.553   0.321   0.252   0.427
B G  4P23D                 10    0.302  23.799  -0.721   0.307   0.165   0.527
B G  4P23D                 11    0.147  11.608  -1.688   0.199   0.152   0.649
E G  4P23D                 12    0.458  36.029  -1.015   0.052   0.084   0.864
E G  4P23D                 13    0.496  39.027  -0.613   0.018   0.047   0.935
E S  4P23D                 14    0.313  36.730  -0.674   0.018   0.019   0.964
E E  4P23D                 15    0.655 114.359  -1.172   0.018   0.047   0.935
E R  4P23D                 16    0.298  68.219  -1.320   0.018   0.088   0.893
B H  4P23D                 17    0.199  36.198  -0.059   0.004   0.420   0.576
B F  4P23D                 18    0.100  20.010   0.886   0.002   0.816   0.182
B V  4P23D                 19    0.100  15.339   0.309   0.001   0.959   0.040
B Y  4P23D                 20    0.058  12.480   1.128   0.001   0.959   0.040
B Q  4P23D                 21    0.062  11.145   0.912   0.001   0.959   0.040
B F  4P23D                 22    0.112  22.559   0.200   0.001   0.959   0.040
B M  4P23D                 23    0.092  18.449   0.037   0.001   0.959   0.040
B G  4P23D                 24    0.058   4.533   0.121   0.001   0.959   0.040
B E  4P23D                 25    0.036   6.237  -0.776   0.001   0.959   0.040
B C  4P23D                 26    0.019   2.668   0.999   0.001   0.959   0.040
B Y  4P23D                 27    0.148  31.692  -0.119   0.001   0.959   0.040
B F  4P23D                 28    0.056  11.159   0.342   0.001   0.959   0.040
B T  4P23D                 29    0.237  32.858   0.541   0.004   0.514   0.481
E D  4P23D                 30    0.668  96.273  -1.101   0.004   0.085   0.910
E G  4P23D                 31    0.754  59.340  -1.930   0.005   0.015   0.979
E T  4P23D                 32    0.482  66.826  -1.828   0.005   0.045   0.951
E Q  4P23D                 33    0.399  71.315  -1.040   0.004   0.420   0.576
E R  4P23D                 34    0.454 104.035   0.365   0.004   0.616   0.381
B I  4P23D                 35    0.128  23.624  -0.324   0.003   0.718   0.279
E R  4P23D                 36    0.297  67.990  -0.011   0.011   0.918   0.071
B Y  4P23D                 37    0.133  28.358  -0.599   0.006   0.962   0.032
B V  4P23D                 38    0.114  17.522   0.101   0.001   0.959   0.040
B T  4P23D                 39    0.159  22.039  -0.315   0.001   0.959   0.040
B R  4P23D                 40    0.121  27.663   0.472   0.006   0.962   0.032
B Y  4P23D                 41    0.114  24.319   0.158   0.001   0.900   0.099
B I  4P23D                 42    0.110  20.313  -0.869   0.002   0.816   0.182
B Y  4P23D                 43    0.203  43.338  -0.168   0.003   0.718   0.279
E N  4P23D                 44    0.416  60.844  -0.343   0.005   0.336   0.660
E R  4P23D                 45    0.374  85.623  -0.866   0.056   0.142   0.802
E E  4P23D                 46    0.503  87.909  -0.231   0.069   0.386   0.545
E E  4P23D                 47    0.328  57.267  -0.420   0.023   0.655   0.322
B Y  4P23D                 48    0.209  44.621   0.723   0.085   0.775   0.140
B V  4P23D                 49    0.168  25.806  -0.496   0.085   0.775   0.140
B R  4P23D                 50    0.260  59.586  -0.418   0.085   0.775   0.140
B Y  4P23D                 51    0.130  27.802  -0.136   0.085   0.775   0.140
E D  4P23D                 52    0.304  43.821  -0.334   0.087   0.683   0.230
B S  4P23D                 53    0.263  30.859  -1.315   0.066   0.296   0.638
E D  4P23D                 54    0.557  80.249  -1.330   0.064   0.216   0.721
E V  4P23D                 55    0.417  64.078  -0.824   0.056   0.142   0.802
E G  4P23D                 56    0.451  35.525  -1.093   0.019   0.141   0.840
E E  4P23D                 57    0.503  87.857  -0.661   0.021   0.279   0.699
B H  4P23D                 58    0.223  40.582  -0.180   0.021   0.451   0.528
E R  4P23D                 59    0.354  80.974  -0.247   0.021   0.451   0.528
B A  4P23D                 60    0.201  22.139  -1.800   0.021   0.279   0.699
B V  4P23D                 61    0.244  37.441  -1.110   0.056   0.142   0.802
E T  4P23D                 62    0.377  52.332  -0.642   0.113   0.043   0.844
E E  4P23D                 63    0.380  66.438  -0.944   0.354   0.048   0.598
E L  4P23D                 64    0.351  64.305  -0.740   0.561   0.047   0.393
B G  4P23D                 65    0.248  19.541  -1.754   0.561   0.047   0.393
E R  4P23D                 66    0.350  80.242  -0.463   0.502   0.102   0.396
E P  4P23D                 67    0.324  45.905  -0.569   0.538   0.173   0.289
B D  4P23D                 68    0.239  34.397  -0.792   0.428   0.171   0.402
E A  4P23D                 69    0.328  36.135  -0.472   0.428   0.171   0.402
E E  4P23D                 70    0.450  78.702  -0.544   0.538   0.173   0.289
E Y  4P23D                 71    0.320  68.320  -0.643   0.502   0.102   0.396
E W  4P23D                 72    0.323  77.754  -0.770   0.455   0.046   0.498
E N  4P23D                 73    0.480  70.272  -1.361   0.354   0.048   0.598
E S  4P23D                 74    0.361  42.309  -0.871   0.278   0.093   0.628
E Q  4P23D                 75    0.327  58.438  -0.805   0.199   0.152   0.649
E P  4P23D                 76    0.314  44.528  -1.143   0.216   0.235   0.548
E E  4P23D                 77    0.331  57.896  -1.296   0.231   0.330   0.439
B I  4P23D                 78    0.205  37.833  -0.687   0.231   0.330   0.439
E L  4P23D                 79    0.306  56.029  -0.744   0.216   0.235   0.548
E E  4P23D                 80    0.331  57.913  -0.919   0.307   0.165   0.527
E R  4P23D                 81    0.331  75.845  -0.884   0.278   0.093   0.628
E T  4P23D                 82    0.375  52.012  -0.993   0.307   0.165   0.527
E R  4P23D                 83    0.342  78.318  -0.168   0.321   0.252   0.427
E A  4P23D                 84    0.362  39.936  -0.502   0.216   0.235   0.548
E E  4P23D                 85    0.314  54.856  -0.702   0.199   0.152   0.649
E L  4P23D                 86    0.411  75.309  -0.718   0.199   0.152   0.649
E D  4P23D                 87    0.416  60.003  -0.709   0.118   0.150   0.732
E T  4P23D                 88    0.486  67.478  -0.920   0.052   0.084   0.864
E V  4P23D                 89    0.301  46.310  -0.598   0.056   0.142   0.802
B C  4P23D                 90    0.264  36.995  -0.457   0.064   0.216   0.721
B R  4P23D                 91    0.272  62.219  -0.697   0.064   0.216   0.721
E H  4P23D                 92    0.369  67.048  -1.084   0.125   0.227   0.648
E N  4P23D                 93    0.336  49.249  -0.900   0.199   0.152   0.649
E Y  4P23D                 94    0.354  75.650  -1.245   0.191   0.086   0.723
E E  4P23D                 95    0.416  72.693  -1.217   0.199   0.152   0.649
E G  4P23D                 96    0.410  32.298  -0.872   0.216   0.235   0.548
B P  4P23D                 97    0.267  37.845  -0.540   0.125   0.227   0.648
E E  4P23D                 98    0.347  60.534  -0.946   0.125   0.227   0.648
E T  4P23D                 99    0.353  48.906  -0.770   0.125   0.227   0.648
E H  4P23D                100    0.374  68.012  -1.406   0.118   0.150   0.732
E T  4P23D                101    0.350  48.559  -1.031   0.118   0.150   0.732
E S  4P23D                102    0.491  57.580  -1.132   0.118   0.150   0.732
E L  4P23D                103    0.420  76.975  -1.093   0.113   0.087   0.800
E R  4P23D                104    0.368  84.341  -0.972   0.113   0.087   0.800
E R  4P23D                105    0.414  94.714  -1.218   0.056   0.142   0.802
E L  4P23D                106    0.507  92.905  -0.798   0.056   0.142   0.802
E E  4P23D                107    0.438  76.606  -0.913   0.020   0.205   0.775
E Q  4P23D                108    0.500  89.336  -1.560   0.021   0.279   0.699
E P  4P23D                109    0.447  63.373  -1.115   0.004   0.197   0.799
E N  4P23D                110    0.435  63.743  -0.467   0.005   0.262   0.733
B V  4P23D                111    0.254  39.071  -1.171   0.004   0.616   0.381
E V  4P23D                112    0.315  48.477  -0.494   0.002   0.816   0.182
B I  4P23D                113    0.257  47.564  -0.711   0.001   0.900   0.099
B S  4P23D                114    0.186  21.799  -1.422   0.001   0.900   0.099
B L  4P23D                115    0.234  42.864  -1.249   0.002   0.816   0.182
B S  4P23D                116    0.294  34.422  -1.017   0.021   0.756   0.223
E R  4P23D                117    0.428  97.943  -1.313   0.022   0.552   0.426
E T  4P23D                118    0.394  54.592  -1.104   0.022   0.552   0.426
E E  4P23D                119    0.450  78.685  -2.023   0.021   0.279   0.699
E A  4P23D                120    0.576  63.475  -1.598   0.018   0.088   0.893
E L  4P23D                121    0.480  87.815  -1.962   0.018   0.019   0.964
E N  4P23D                122    0.740 108.307  -1.134   0.005   0.015   0.979
E H  4P23D                123    0.529  96.152  -1.416   0.005   0.045   0.951
E H  4P23D                124    0.437  79.418   0.196   0.005   0.262   0.733
B N  4P23D                125    0.102  14.918  -0.196   0.003   0.718   0.279
B T  4P23D                126    0.229  31.762   1.244   0.001   0.900   0.099
B L  4P23D                127    0.020   3.607   1.078   0.001   0.900   0.099
B V  4P23D                128    0.063   9.622   0.970   0.001   0.959   0.040
B C  4P23D                129    0.015   2.064   1.320   0.001   0.959   0.040
B S  4P23D                130    0.115  13.537   0.794   0.001   0.959   0.040
B V  4P23D                131    0.023   3.535   0.192   0.001   0.900   0.099
B T  4P23D                132    0.248  34.453  -0.375   0.004   0.514   0.481
E D  4P23D                133    0.422  60.839  -0.929   0.005   0.262   0.733
B F  4P23D                134    0.164  32.955  -0.986   0.004   0.197   0.799
E Y  4P23D                135    0.313  66.952  -0.150   0.004   0.197   0.799
B P  4P23D                136    0.196  27.770  -0.666   0.004   0.138   0.858
E A  4P23D                137    0.424  46.703  -0.583   0.004   0.197   0.799
E K  4P23D                138    0.460  94.663  -0.152   0.004   0.616   0.381
B I  4P23D                139    0.028   5.106   0.203   0.001   0.900   0.099
E K  4P23D                140    0.297  61.072   1.046   0.001   0.959   0.040
B V  4P23D                141    0.029   4.473   0.338   0.001   0.959   0.040
B R  4P23D                142    0.244  55.968   1.668   0.001   0.959   0.040
B W  4P23D                143    0.064  15.416   0.785   0.001   0.959   0.040
E F  4P23D                144    0.289  57.962   1.090   0.001   0.959   0.040
E R  4P23D                145    0.275  62.975   0.605   0.002   0.816   0.182
E N  4P23D                146    0.605  88.601  -0.274   0.005   0.045   0.951
E G  4P23D                147    0.678  53.382  -1.184   0.005   0.015   0.979
E Q  4P23D                148    0.608 108.535   0.286   0.004   0.514   0.481
E E  4P23D                149    0.675 117.853   0.404   0.004   0.616   0.381
E E  4P23D                150    0.273  47.623   0.708   0.021   0.451   0.528
E T  4P23D                151    0.466  64.676  -0.592   0.021   0.279   0.699
E V  4P23D                152    0.635  97.599  -0.506   0.019   0.141   0.840
E G  4P23D                153    0.691  54.358  -1.456   0.019   0.141   0.840
E V  4P23D                154    0.304  46.740  -0.435   0.004   0.420   0.576
E S  4P23D                155    0.504  59.116  -0.809   0.004   0.616   0.381
E S  4P23D                156    0.432  50.642  -1.026   0.022   0.552   0.426
E T  4P23D                157    0.363  50.376  -1.871   0.022   0.359   0.619
E Q  4P23D                158    0.509  90.818  -0.788   0.022   0.552   0.426
E L  4P23D                159    0.394  72.233  -0.592   0.023   0.655   0.322
E I  4P23D                160    0.320  59.237  -0.183   0.023   0.655   0.322
E R  4P23D                161    0.451 103.256  -1.097   0.004   0.514   0.481
E N  4P23D                162    0.538  78.763  -0.850   0.005   0.262   0.733
E G  4P23D                163    0.612  48.188  -1.990   0.005   0.045   0.951
E D  4P23D                164    0.674  97.138  -1.681   0.005   0.015   0.979
E W  4P23D                165    0.339  81.602  -0.881   0.004   0.197   0.799
B T  4P23D                166    0.158  21.859   0.239   0.003   0.718   0.279
B F  4P23D                167    0.066  13.186   0.100   0.001   0.900   0.099
B Q  4P23D                168    0.094  16.699   0.251   0.001   0.959   0.040
B V  4P23D                169    0.060   9.222   0.067   0.001   0.959   0.040
B L  4P23D                170    0.121  22.082   0.282   0.001   0.959   0.040
B V  4P23D                171    0.037   5.748  -0.802   0.001   0.959   0.040
B M  4P23D                172    0.134  26.893   0.417   0.001   0.959   0.040
B L  4P23D                173    0.036   6.537  -0.387   0.001   0.959   0.040
E E  4P23D                174    0.277  48.462   0.534   0.001   0.959   0.040
B M  4P23D                175    0.059  11.806   0.640   0.003   0.718   0.279
E T  4P23D                176    0.306  42.470   0.889   0.005   0.262   0.733
E P  4P23D                177    0.373  52.900  -0.356   0.004   0.085   0.910
E R  4P23D                178    0.695 159.155  -0.249   0.005   0.015   0.979
E R  4P23D                179    0.637 145.896   0.069   0.005   0.015   0.979
E G  4P23D                180    0.593  46.685  -0.687   0.005   0.015   0.979
E E  4P23D                181    0.442  77.287   0.198   0.005   0.262   0.733
E V  4P23D                182    0.467  71.793   1.523   0.003   0.718   0.279
B Y  4P23D                183    0.059  12.694   1.035   0.001   0.900   0.099
B T  4P23D                184    0.256  35.535   1.138   0.001   0.959   0.040
B C  4P23D                185    0.017   2.345   1.184   0.001   0.959   0.040
B H  4P23D                186    0.214  38.890   1.567   0.001   0.959   0.040
B V  4P23D                187    0.034   5.287   0.203   0.001   0.959   0.040
E E  4P23D                188    0.386  67.452   0.822   0.001   0.959   0.040
B H  4P23D                189    0.095  17.262   0.160   0.002   0.816   0.182
E P  4P23D                190    0.494  70.141  -0.372   0.004   0.197   0.799
E S  4P23D                191    0.504  59.034  -1.038   0.004   0.085   0.910
B L  4P23D                192    0.198  36.162  -0.036   0.004   0.085   0.910
E K  4P23D                193    0.685 140.822   0.216   0.018   0.047   0.935
E S  4P23D                194    0.527  61.729   0.164   0.005   0.045   0.951
E P  4P23D                195    0.506  71.730  -0.366   0.004   0.197   0.799
B I  4P23D                196    0.175  32.301   0.305   0.004   0.616   0.381
E T  4P23D                197    0.414  57.380   0.362   0.001   0.900   0.099
E V  4P23D                198    0.289  44.435   1.547   0.001   0.900   0.099
E E  4P23D                199    0.509  88.887   1.047   0.001   0.900   0.099
B W  4P23D                200    0.110  26.527   0.612   0.002   0.816   0.182
E R  4P23D                201    0.531 121.599   1.245   0.004   0.616   0.381
E A  4P23D                202    0.351  38.680  -1.399   0.004   0.197   0.799
E Q  4P23D                203    0.807 144.112  -0.117   0.003   0.003   0.994

Explain the output.  Go back.
```

---
